# Variation in malariometric and red cell indices in children in the Mount Cameroon area following enhanced malaria control measures: evidence from a repeated cross-sectional study

**DOI:** 10.1186/1475-2875-13-334

**Published:** 2014-08-26

**Authors:** Irene UN Sumbele, Teh R Ning, Orelien SM Bopda, Theresa Nkuo-Akenji

**Affiliations:** Department of Zoology and Animal Physiology, University of Buea, Buea, Cameroon; Department of Microbiology and Parasitology, University of Buea, Buea, Cameroon

**Keywords:** Malaria, Anaemia, Children, Prevalence, Insecticide-treated nets, Microcytic anaemia

## Abstract

**Background:**

Following enhanced malaria control measures, such as nationwide free distribution of insecticide-treated bed nets (ITN) by the government of Cameroon, its impact on malariometric and red cell indices in children ≤14 years in Muea, in the Mount Cameroon area was evaluated.

**Methods:**

Two cross-sectional studies were conducted during the malaria transmission season (March-July) in 2006 (baseline) and 2013 (follow-up), respectively. The investigative methods included the use of a questionnaire to assess ITN use and coverage, clinical evaluation and laboratory investigations. Blood sample collected from each child was used for the preparation of blood films for detection of malaria parasites and density as well as full blood count determination using standard procedures and also an automated haematology analyzer.

**Results:**

The majority of children (81.5%) possessed an ITN in 2013. The proportion of effective users of ITN increased significantly from 20.9% (CI = 17.3-25%) in 2006 to 35.2% (CI = 31–39.7%) in 2013. The highest relative risk reduction in prevalence during the follow-up study was observed in malaria anaemia (79%, CI = 58.0-69.1% [69.1 to 14.5%]), followed by gametocytaemia (71.6%, CI = 58.9-80.3% [25.6 to 7.3%]), anaemia (64%, CI = 58.0-69.1% [80.1 to 28.9%]), and malaria parasitaemia (57.2%, CI = 51.4-62.3% [85.4 to 36.6%]). In the baseline survey, the prevalence of splenomegaly was significantly highest (*χ*^2^ = 18.3, P <0.001) in the youngest group of children while in the follow-up study, it was highest in the oldest (*χ*^2^ = 6.03, P = 0.049). The overall prevalence of mild, moderate and severe anaemia in the study population at baseline (59.6, 14.9, 6.3%) decreased significantly (P <0.001) to 24.4, 2.7 and 1.3%, respectively during the follow-up with the highest relative risk reduction in prevalence occurring in moderate anaemia (82.1%, CI = 67.3-90.2% [14.9 to 2.7%]). Microcytic anaemia also decreased significantly (P <0.001) from 56 to 7.7% during the follow-up survey.

**Conclusion:**

Following interventions, anaemia (moderate to severe) was a more sensitive measure to changes in malaria exposure and children between 11–14 years of age experienced a significant increase in malaria-related morbidity.

## Background

The estimates of malaria burden are based on malariometric indices, such as prevalence of malaria parasitaemia, spleen rate and anaemia in defined risk groups [1]. Childhood anaemia may be a useful indicator of the burden of malaria and of the progress in malaria control [2]. As an intra-erythrocytic parasite of blood, *Plasmodium* induces haematological alterations. It is essential to monitor these alterations in order to assess the impact of malaria control interventions.

There are many preventive measures of malaria both at community and individual level, with the most promising being insecticide-treated bed nets (ITNs) [3–5]. The use of ITNs may be easier to implement and sustain than available alternative vector control methods, such as environmental sanitation. This is because of the immediate perceptible benefits to the user in personally preventing them from the nuisance of mosquitoes, as well as their killing effect on head lice [6] and bed bugs [7]. In order to be protected, individuals must not only own ITNs but also use them. As high coverage rates are needed to realize the full potential of vector control, the World Health Organization (WHO) recommends that in areas targeted for malaria prevention, and for which ITNs are selected as the vector control method, they should be made available to all people at risk, i.e., universal access [8]. In Cameroon, a high (>70%) percentage of the population potentially covered by ITNs distributed in 2011 was reported, yet no decrease in admissions and deaths were registered [9].

A strong association between interventions and their impact on malaria morbidity and mortality has been reported in Sao Tome and Principe [9]. When full coverage is achieved, ITNs have been reported to reduce clinical episodes of malaria caused by *Plasmodium falciparum* and *Plasmodium vivax* infection by 50% on average (range 39-62%), as well as reducing the prevalence of high density parasitaemia [3]. Despite efforts made by the National Malaria Control Programme (NMCP) to curb the disease burden, the prevalence is seemingly not on the increase but still a public health problem. Following substantial investments by the government of Cameroon in malaria control, coupled with the lack of population-based surveys or specific studies of the impact of interventions, the need for evaluation of intervention is critical. The objective of this study was to evaluate the impact of enhanced malaria control measures by the free distribution of ITNs on malariometric and red cell indices in children ≤14 years in Muea, in the Mount Cameroon area.

## Methods

### Study area and participants

The study was carried out in Muea village, a semi-rural setting in the Mount Cameroon area. The coordinates of the study area ranged from altitude 540 m, latitude 04°10.464′N, longitude 009°18.168′E to 556 m, 04°10.015′N and 009°18.009′E. The study area has been described in detail by Sumbele *et al.* [10]. The children from the community who participated in the study weighed >5 kg, were ≤14 years old, free from other clinical conditions not related to malaria and sickle test negative. Children who presented with fever, joint pains, headache, malaise, abdominal pain, nausea, and vomiting were considered to be symptomatic (clinical malaria).

### Study design

**Figure 1:**
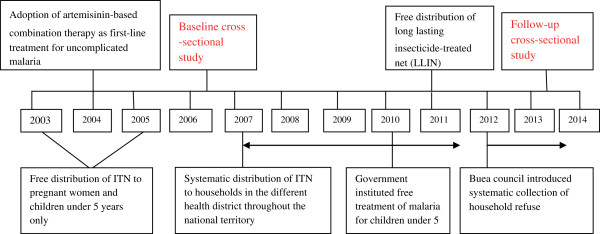
**Relevant malaria control interventions in Cameroon.**

Following several control programmes, including the free distribution of ITNs (Figure 1) by the government of Cameroon through the NMCP, two cross-sectional studies were conducted during the malaria transmission season (March-July) in Muea, a semi-rural community in the Mount Cameroon area. The first cross-sectional study (baseline) was carried out in 2006 following the free distribution of ITNs to malaria-vulnerable groups (pregnant women and children under five years) only, in selected regions of the country. A second cross-sectional study (follow-up) was conducted in 2013 following an upscale systematic distribution of ITN and long-lasting, insecticidal nets (LLINs) to all households in all health districts and the institution of free malaria treatment for children under five years of age.

At the start of each study, the parents, guardians and children were educated at their various neighbourhoods. During education, the study protocol was explained and the benefits of participating in the study highlighted. Children were enrolled into the study only when parent or guardian signed the assent form or gave a verbal consent. Thereafter the children were clinically evaluated and their parent/caregivers interviewed using a semi-structured questionnaire to obtain information on ITN possession and use. Blood samples were collected for thick and thin smear microscopy as well as a standard full blood count. Labelled blood samples were transported on ice in a cool box of temperature between 8-10°C to the University of Buea Malaria Research Laboratory for further analyses.

### Questionnaire

A semi-structured questionnaire was administered to the participant/parent/guardians/caregiver to obtain information about ITN/LLINs use, its state, various malaria control measures and occurrence of malaria since they started using the nets. Children, who had good nets (potent ITN/LLINs, without hole/tears) that were used regularly, were considered effective users while children who had good nets but used them irregularly or had torn nets were considered as ineffective users.

### Clinical evaluation

The clinical evaluation carried out by trained medical personnel in their homes consisted in part by the measurement of body temperature and examination of the spleen. Axillary body temperature was measured using a digital thermometer. A child with a body temperature ≥37.5°C was considered febrile. Ages of the children were obtained from their mothers and verified from their birth certificates. The tip of the spleen was felt by pressing the abdomen under the left coastal border and splenomegaly was graded according to the classification of Hackett [11].

### Laboratory methods

Approximately 4–5 ml of blood sample was collected from the children by venipuncture into 5 ml sterile disposable syringes and dispensed into micro-containers or vacutainers containing ethylenediaminetetraacetate (EDTA) solution. Drops of whole blood were dispensed immediately on slides to prepare blood films. The thick and thin blood smears prepared on glass slides at the time of blood sampling were stained with Giemsa and examined following standard protocols [12]. Parasite density was determined on the basis of number of parasites per 200 leukocytes on thick blood film with reference to subjects’ white blood cell counts (WBC). If gametocytes were seen, the count was extended to 500 leukocytes [13]. In 2006, haemoglobin (Hb) concentration was measured in the field using a Stanbio STAT-Site^R^ Test Kit (STAT-site M^Hgb^ Meter, Stanbio Laboratory, Texas, USA) following the manufacturer’s instructions. Also WBC and red blood cell counts (RBC) were determined using the improved Neubauer haemocytometer as described by Cheesbrough [12] and Dacie and Lewis [14]. In 2013, a complete blood count: RBC, WBC, Hb, haematocrit (Hct), platelet, mean corpuscular volume (MCV), mean corpuscular haemoglobin (MCH), and mean corpuscular haemoglobin concentration (MCHC) was run following the manufacturer’s instructions using an automated haematology analyzer, the Beckman Coulter counter (URIT 3000).

### Definitions and end points

-  Fever was defined as temperature ≥37.5°C;
-  Asymptomatic malaria (AM) was defined as the presence of *Plasmodium* with an axillary temperature of <37.5°C;
-  Clinical malaria (CM) was defined as the presence of any species of *Plasmodium*, with an axillary temperature of ≥37.5°C or reported fever in previous 48 hours;
-  Parasitaemia was categorized as low (<1,000 parasite/μL blood), moderate (1,000-4,999 parasites/μL blood) and high (≥5,000 parasites/μL blood);
-  A Hb level of <11 g/dL was classified as anaemic and further categorized as mild (Hb between 10.1 and 10.9 g/dL), moderate (Hb between 7.0 and 10.0 g/dL) and severe (Hb <7 g/dL) anaemia [12, 15];
-  Malarial anaemia (MA) was defined as children with Hb <11 g/dL and malaria parasite positive [16];
-  Microcytosis was defined as MCV of less than 73 fl [17];
-  Hypochromasia was defined as a MCHC of less than 320 g/L [18].

### Data analysis

Data were entered into spreadsheets using Microsoft Excel and analysed with the IBM Statistical package for Social Sciences (SPSS) version 19 (SPSS Inc., Chicago, IL, USA). Data were summarized into means and standard deviations (SD), and percentages were used in the evaluation of the descriptive statistics. Proportions were compared using the Chi-square test (χ^2^). Parasitaemia was log transformed before analysis. Means were compared using independent sample t-test and analysis of variance (ANOVA) where appropriate. The 95% confidence interval (CI) for the difference between two proportions and relative risk reduction were calculated using a Microsoft Excel confidence interval calculator as described by the Newcombe-Wilson method [19, 20]. The relative risk reduction and its confidence limits were obtained as one minus the relative risk and the values presented as percentages (%) by multiplying by a hundred (100).

### Ethical considerations

Before commencement of each study, administrative clearance was obtained from the South West Regional Delegation of Public Health. The institutional review board hosted by the Faculty of Health Sciences, University of Buea issued the ethical clearance document. Additional authorization was obtained from the local health committee and the village chief. Children participated in the study if a parent or guardian signed the informed consent/assent form. The parents or guardians and their children were informed that their participation in the study was voluntary and they could withdraw at any time without explanation.

## Results

### Characteristics of study populations

In 2006, a total of 411 children (215, 52.3% males and 196, 47.7% females) ≤14 years with a mean age of 6.4 (7.4) years were examined for the presence of malaria parasites and some malaria-related indices. Two-hundred and twenty (53.5%) of the children were age ≤ five years while 134 (32.6%) and 57 (13.9%) belonged to the six to ten and 11–14 years age groups, respectively. A total of 129 (31.4%, CI = 27.1-36%) children possessed or used an ITN. Only 86 (20.9%, CI = 17.3-25%) of them used them effectively. The proportion of effective users in the different age groups was comparable (P = 0.82).

In the follow-up study in 2013, 454 children (211, 46.5% males and 243, 53.5% females) ≤14 years with a mean age of 6.7 (3.4) years were examined. Of these, 177 (39.0%), 205 (45.2%) and 72 (15.9%) of the children were in the ≤ five, six to ten and 11–14 years age groups, respectively. The majority of the children (81.5%, CI = 77.7-84.8%) in the community possessed or used an ITN. The proportion of effective users of ITN in 2013 increased significantly from 20.9% (86, CI = 17.3-25%) in 2006 to 35.2% (160, CI = 31–39.7%). The proportion of effective users was significantly highest (χ2 = 9.52, P = 0.01) in the < five years age group (42.4%, CI = 35.3-49.7%) when compared with the six to ten (33.7%, CI = 27.5-40.4%) and the 11–14 years (22.2%, CI = 14.2-33.1%) age groups.

### Variation in malariometric indices

The prevalence of fever, malaria parasitaemia, gametocytaemia, splenomegaly, CM, AM, anaemia, and MA decreased significantly (P <0.05) in 2013 when compared with the baseline data in 2006 (Table 1). In decreasing order, the highest relative risk reduction in prevalence during the follow-up study in 2013 was observed in MA (79%, CI = 58.0-69.1%), where MA prevalence decreased from 69.1 to 14.5%. This was followed by gametocytaemia (71.6%), anaemia (64%), CM (62.2%), AM (60.6%), malaria parasitaemia (57.2%), and splenomegaly (39.3%) as shown on Table 1. The prevalence of fever showed the least relative risk reduction in prevalence (23.5%).

**Table 1:** **Variation in prevalence of malariometric indices**

Variable		Baseline (2006)	Follow-up (2013)	χ2 P-value	Relative risk reduction	Odds ratio
Fever	% (n)	28.2 (116)	21.6 (98)	0.03^a^	23.5	0.7
	CI	24.1-32.8	18.1-25.6		3.4-39.4	0.51-0.96
Malaria parasitaemia	% (n)	85.4 (351)	36.6 (166)	< 0.001^c^	57.2	0.10
	CI	81.7-88.5	32.3-41.1		51.4-62.3	0.07-0.14
Gametocytaemia	% (n)	25.6 (105)	7.3 (33)	< 0.001^c^	71.6	0.23
	CI	21.6-30.0	5.2-10.0		58.9-80.3	0.15-0.35
Splenomegaly	% (n)	23.6 (97)	14.3 (65)	< 0.001^c^	39.3	0.54
	CI	19.8-27.9	11.4-17.8		19.4-54.4	0.38-0.77
CM	% (n)	25.1 (103)	9.5 (43)	< 0.001^c^	62.2	0.31
	CI	21.1-29.5	7.1-12.5		47.4-72.8	0.21-0.46
AM	% (n)	60.3 (248)	23.8 (108)	< 0.001^c^	60.6	0.25
	CI	55.5-65.0	20.1-27.9		52.7-67.2	0.15-0.28
Anaemia	% (n)	80.1 (329)	28.9 (131)	< 0.001^c^	64	0.10
	CI	75.9-83.6	24.9-33.2		58.0-69.1	0.07-0.14
MA	% (n)	69.1 (284)	14.5 (66)	< 0.001^c^	79	0.08
	CI	64.5-73.4	11.6-18.1		73.5-83.3	0.05-0.12

^a^Significant at P <0.05 level, ^c^significant at P <0.001 level.

The trends in prevalence of fever and gametocytaemia in the different age groups were comparable at baseline and in the follow-up study. Although not statistically significant, the highest prevalence of malaria parasite was observed in children between 11–14 years of age in the follow-up study (40.3%, CI = 29.7-51.8%), while in the baseline study children in the six to ten years age group had the highest prevalence (88.1%, CI = 81.5-92.5%) as indicated in Table 2.

**Table 2:** **Effect of age on the prevalence of malariometric indices**

Variable	Period	Age groups in years			P-value
		≤5% (n)	6-10% (n)	11-14% (n)	
Age group distribution	Baseline	53.5 (220)	32.6 (134)	13.9 (57)	
	Follow-up	39.0 (177)	45.2 (205)	15.9 (72)	
Fever	Baseline	26.8 (59)	32.8 (44)	22.8 (13)	0.29
	Follow-up	20.9 (37)	23.0 (47)	19.4 (14)	0.78
Malaria parasitaemia	Baseline	84.5 (186)	88.1 (118)	82.5 (47)	0.53
	Follow-up	37.3 (66)	34.6 (71)	40.3 (29)	0.67
Gametocytaemia	Baseline	29.1 (64)	23.1 (31)	17.5 (10)	0.15
	Follow-up	9.0 (16)	6.8 (14)	4.2 (3)	0.39
Splenomegaly	Baseline	31.8 (70)	15.7 (21)	10.5 (6)	<0.001^c^
	Follow-up	12.4 (22)	12.7 (26)	23.6 (17)	0.049
CM	Baseline	23.6 (52)	29.1 (39)	21.1 (12)	0.39
	Follow-up	10.7 (19)	8.8 (18)	8.3 (6)	0.76
AM	Baseline	60.9 (134)	59.0 (79)	61.4 (35)	0.92
	Follow-up	22.6 (40)	22.4 (46)	30.6 (22)	0.34
Anaemia	Baseline	84.1 (185)	78.4 (105)	68.4 (39)	0.03^a^
	Follow-up	37.9 (67)	28.8 (59)	6.9 (5)	<0.001^c^
MA	Baseline	73.2 (161)	66.4 (89)	59.6 (34)	0.103
	Follow-up	20.3 (36)	14.1 (29)	1.4 (1)	<0.001^c^

^a^Significant at P <0.05 level, ^c^significant at P <0.001 level.

In the baseline survey, the prevalence of splenomegaly was significantly highest (χ^2^ = 18.3, P <0.001) in the youngest group of children while in the follow-up study, the highest prevalence of splenomegaly that approached significance (χ^2^ = 6.03, P = 0.049) was observed in the oldest group of children (Table 2). The prevalence of splenomegaly in children in the 11–14 years age group was higher (χ^2^ = 3.71, P = 0.054) in the follow-up study (23.6%, CI = 15.3-36.5%) than at the baseline (10.5%, CI = 4.9-21.1%) with an odds ratio of 2.63 (CI = 0.96-7.18).

The prevalence of anaemia in both study periods decreased significantly (P <0.05) with increased age (Table 2). The highest relative risk reduction in prevalence occurred in the 11–14 years age group (89.9%, CI = 75.9-95.7%) when compared with the < five years (55.0%, CI = 45.2-63.1%) and six to ten years (63.3%, 53.6-70.9%) age groups.

At baseline a comparison of MA prevalence within the age groups showed no significant difference (P >0.05) while in 2013, the prevalence decreased significantly (P <0.001) with an increase in the age group with children in the 11–14 years age group having a 97.7% (CI = 83.5-99.7%) relative risk reduction in prevalence (Table 2).

Although not statistically significant, the asexual geometric mean parasite density (GMPD)/μL of blood in 2013 was higher (834.8) than the baseline value (789.9). As shown in Table 3, the decrease in GMPD with increased age was statistically significant (P <0.01) at baseline, while in the follow-up study, no significant difference in neither GMPD with age group nor a trend was observed.

**Table 3:** **Comparison of geometric mean parasite density per μL of blood within the age groups and study period**

Age groups in years	Baseline (2006)			Follow-up (2013)		
	N	GMPD	ANOVA	N	GMPD	ANOVA
			P- value			P- Value
≤5	186	1,003.4		62	1076.5	
6-10	118	637.4	5.54	69	639.0	1.42
11-14	47	524.9	0.004^§b^	30	832.7	0.244^§^
Total	351	789.9		161	834.8	

^§^Log of parasite counts used in the analysis, ^b^Significant at P <0.01 level.

The disparity in prevalence of low, moderate and high asexual malaria parasite at baseline (60.1%, CI = 54.9-65.1% [210]; 27.6%, CI = 23.2-32.5% [97]; 12.3%, CI = 9.2-16.1% [43]) and follow-up (56.5%, CI = 48.8-63.9% [91]; 29.8%, CI = 23.3-37.3% [48] and 13.7% CI = 9.2-19.8% [22], respectively) was not significant (P = 0.74).

### Variation in red cell indices

Between the baseline survey and the follow-up study, the overall mean Hb concentration significantly increased (t = 17.08, P <0.001) from 9.5 (1.7) g/dL (CI = 9.3-9.7 g/dL) to 11.6 (1.9) g/dL (CI = 11.4-11.8 g/dL) with a mean difference of 2.1 g/dL (CI = 1.9-2.4 g/dL). Children who were malaria parasite positive in the follow-up study had significantly higher (P <0.001) mean Hb, Hct, MCH, MCHC, and MCV when compared with their baseline counterpart. On the other hand, the mean RBC was significantly lower in malaria parasite positive children in the follow-up study than those at baseline (Table 4).

**Table 4:** **A comparison of mean red cell indices in malaria parasite positive children at baseline and follow-up**

Variable	Period	N	Mean (SD)	t-test	Mean difference
				P-value	CI
Hb (g/dL)	Baseline	351	9.4 (1.8)	10.80	1.8
	Follow-up	161	11.3 (1.9)	<0.001^c^	1.5-2.2
Hct (%)	Baseline	351	31.3 (5.0)	5.94	2.9
	Follow-up	161	34.2 (5.3)	<0.001^c^	1.9-3.9
MCH (pg)	Baseline	333	19.0 (7.8)	9.86	6.6
	Follow-up	140	25.6 (2.4)	<0.001^c^	5.3-7.9
MCHC (g/dL)	Baseline	334	304.8 (58.3)	4.66	23.2
	Follow-up	140	328.0 (14.8)	<0.001^c^	13.4-33.1
MCV (fl)	Baseline	334	64.7 (32.1)	4.92	13.5
	Follow-up	140	78.2 (6.2)	<0.001^c^	8.1-18.9
RBC × 10^12^/L	Baseline	328	5.7 (2.0)	-6.4	-1.1
	Follow-up	138	4.5 (0.7)	<0.001^c^	- 1.5 - -0.8

^c^significant at P <0.001 level.

The overall prevalence of mild, moderate and severe anaemia in the study population at baseline (59.6, 14.9, 6.3%) decreased significantly (P <0.001) to 24.4, 2.7 and 1.3%, respectively during follow-up as shown in Table 5. The highest relative risk reduction in prevalence of anaemia was observed in moderate anaemia (82.1%, CI = 67.3-90.2% [14.9 to 2.7%]).

**Figure 2:**
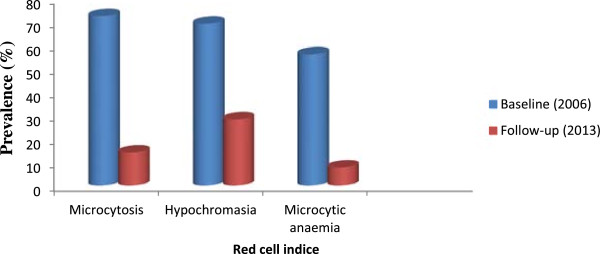
**Prevalence of microcytosis, hypochromasia and microcytic anaemia at baseline and during follow-up.**

The prevalence of microcytosis, hypochromasia and microcytic anaemia (MCV <73 fl and Hb <11 g/dL) decreased significantly (P <0.001) from >55% in 2006 to <29% in 2013 (Figure 2). The occurrence of microcytic anaemia in children in the follow-up survey had the highest reduction in relative risk (86.3%, CI = 80.5-90.3%) when compared with microcytosis (80.5%, CI = 75–84.8%) and hypochromasia (59.1%, CI = 51.6-65.4%).

**Table 5:** **Variation in the different categories of anaemia in the study populations**

Anaemia category		Period prevalence		Relative risk reduction (%)	Odds ratio
		Baseline (2006)	Follow-up (2013)		
Mild	% (n)	59.6 (242)	24.4 (110)	58.7	0.22
	CI	54.2-63.7	20.7-28.6	50.5-65.5	0.17-0.3
Moderate	% (n)	14.9 (61)	2.7 (12)	82.1	0.16
	CI	11.8-18.7	1.53-4.6	67.3-90.2	0.08-0.30
Severe	% (n)	6.3 (26)	1.3 (6)	79.0	0.20
	CI	4.4-9.13	0.6-2.9	49.6-91.3	0.08-0.49

## Discussion

Most parts of Cameroon have approached or met the Roll Back Malaria (RBM) household ITN coverage of at least 60% as reported by previous studies [9]. However, malaria still remains a public health concern even though LLINs were distributed freely in 2011. This study aimed at assessing the impact of ITN use as one of the enhanced malaria control measures on malariometric and red cell indices in children in Muea, in the Mount Cameroon area.

Parasitaemia is one of the indices used in estimating the burden of malaria and the impact of interventions. The prevalence of malaria parasitaemia is on the decline in the Mount Cameroon area as revealed by the study. Overall, the prevalence of malaria parasite obtained in the follow-up study (36.6%) was lower than those obtained at baseline (85.4%) and a similar study conducted in 2012 (44.3%) in this part of the Mount Cameroon area by Kimbi *et al*. [21]. The achievement of a relative risk reduction in malaria parasite prevalence of 57.2% may be credited to all the control efforts implemented by the Cameroon government, through the NMCP in the Ministry of Public Health. Some of the measures included the free distribution of LLINs to all age groups, as well as free treatment of malaria among children less than five years of age. Also, the government introduced several educative health campaigns on malaria prevention in public and private media (radio and television channels). Such messages included the recognition of common signs and symptoms of malaria and the need to get prompt and effective treatment following diagnosis. Other protective measures encouraged included eliminating breeding sites by drainage of pools in which water collects, regular collection of household refuse by designated authorities to reduce container breeding of mosquitoes, filling up of pot-holes, chemical killing of mosquito larvae in well defined and limited breeding sites and compulsory monthly clean up of the environment by communities.

The study brings to light a change in morbidity in children in the follow-up survey. Although not statistically significant, the asexual GMPD/μL of blood in 2013 was higher (834.8) than the baseline value (789.9). Furthermore, in the follow-up study, children in the 11–14 years age group had a significantly higher (P = 0.04) GMPD/μL of blood than their baseline equivalent. The highest prevalence of malaria parasite and the significant increase in parasite density observed in children 11–14 years old, unlike in the baseline study, probably reflects the manner of usage of ITNs by children in the study area. Results from the study indicate that while the effective use of ITNs in 2006 was comparable in the different age groups, in 2013 children in the 11–14 years age group registered a significant decrease in usage. Consequently, they were more exposed to infective mosquito bites than the other age groups. This may have resulted in the high prevalence and density of malaria parasite observed in this age group. Hence, children in the 11–14 years age group most likely did not acquire immunity to parasitization *per se* but may have acquired immunity that prevented the development of febrile disease as the study revealed an increase in asymptomatic malaria in the age group (Table 2). This is in line with Bejon *et al*. [22] who reported a shift away from febrile malaria to acquiring asymptomatic parasitaemia with increasing age. The asymptomatic parasitaemia may be beneficial in inducing and sustaining partial immunity against malaria [23], although a significant drawback is the suppression of Hct levels [24].

It is not surprising that children under five years recorded the highest parasitaemia when compared with the older children in spite of their effective use of ITNs. Children under five years are more vulnerable to the disease in areas of high transmission [21, 25] as naturally acquired immunity builds up in older children following repeated exposure to the parasite. In another study [22], the use of ITNs was associated with remaining infection-free rather than acquiring asymptomatic infection or febrile malaria. The high effective use of ITNs in this age group, culminating in a lack of exposure to infective mosquito bites, could have prevented the development of blood stage immunity, the protective effect of living in a high-transmission area [22], hence, the high parasitaemia observed.

The prevalence of *Plasmodium* gametocytes, the sexual, non-proliferating blood stage in the life cycle, significantly reduced in the follow-up study. It is plausible that the implementation of a highly gametocytocidal, artemisinin-based combination therapy (ACT) [26], namely amodiaquine-artesunate, as first-line treatment for uncomplicated malaria by the government of Cameroon, may have contributed to the reduction in gametocyte prevalence. However, malaria transmission is dependent on mosquito vector dynamics, the proportion of humans with peripheral gametocytaemia, and the infectiousness of circulating gametocytes to mosquitoes. The reduction in prevalence of peripheral gametocytaemia observed in the follow-up survey is of utmost importance as this probably attests to a decline in malaria transmission following a reduction in the infectiousness of *Anopheles* mosquitoes.

Splenomegaly is the main clinical marker of endemicity in *P. falciparum* transmission areas [27]. The minimal reduction in the prevalence of splenomegaly when compared with other malariometric indices indicates malaria is still *meso*-endemic [28] in the study area. Worthy of note is the significant change in morbidity with age when both surveys were compared. Contrary to the baseline trend, the follow-up study showed an increase in splenomegaly prevalence with age. Persistent parasitaemia from treatment failure [29] and a pronounced immunological response especially in children ≤ five years [25] are known to contribute to splenic enlargement. However, with the introduction of ACT as the first-line treatment for malaria in Cameroon, the widespread use of ITNs and prompt access to effective treatment in children, especially those aged under five, the spleen rate is expected to decline. While the increased prevalence of splenomegaly in children age over ten years may be the consequence of increased presence of malaria parasite and density in this age group, the lowered spleen rate in children under five years may be ascribed to the benefits of the therapeutic actions of ACT and the effective use of ITNs. ACT is given in all hospitals in Cameroon to children aged under five with malaria, free of cost, a policy the government instituted in 2010. The beneficial features of ACT include rapid and substantial reduction of the parasite biomass, rapid parasite clearance and effective action against multidrug resistant *P. falciparum* [30], thus preventing recrudescence and multiple infections which may over work the spleen, consequently leading to splenomegaly. Studies indicate that the spleen-specific pitting function accounts for a large fraction of parasite clearance in artemisinin-treated patients [31].

The increase in mean Hb concentration from 9.4 (1.8) in the baseline study to 11.3 (1.9) g/dL in the follow-up study, regardless of the status, signifies a great improvement in the health of children in this study area. WHO and RBM recommend that anaemia be used as an additional indicator to monitor malaria burden at community level as interventions are nationally scaled-up [32]. The significant reduction in anaemia and MA at follow-up suggests a substantial diminution in malaria-related morbidity in the community. On the other hand, the significant decline in morbidity related to malaria is not unexpected in a community which benefits from free ITNs, LLINs and ACT in the vulnerable group. In addition, non-malaria-related intervention, such as regular deworming of school-age children carried out by the health authorities and parents, may have contributed to the improved Hb levels and health of the children. Furthermore, the acquisition of knowledge by caregivers/parents on the health and nutritional status of their children following the education on the outcome of the baseline study, may have played a significant role in improving health and well being in the community.

Even though both studies had as a limitation a small number of children in the 11–14 years age group, it is unlikely that the difference in numbers may have introduced a bias as the mean difference in age was of no consequence. The higher prevalence of anaemia in the ≤ five years group with decreasing severity with age is in line with previous studies that anaemia due to malaria is more severe in younger children in areas of intense transmission [25]. Children in this age group are more vulnerable to infection with malaria than others with severe and potentially fatal complications.

Studies by Korenromp *et al*. [2] reported the impact of ITNs on anaemia to be more pronounced than that for parasitaemia prevalence and CM, with moderate to severe anaemia being a more sensitive measure to changes in malaria exposure due to increasing coverage of malaria interventions. Findings from the study attest to this as the highest relative risk reduction in prevalence was observed in moderate and severe anaemia indicating that the nationwide free distribution of ITNs and use had a major impact on the health of the children. Nonetheless, although the proportion of effective users of ITNs in the population was low, the non-effective users probably benefitted from the reduction in mosquito vector population achieved in the community from nets of effective users, hence the reduction in anaemia and severity.

Results from the baseline survey indicated a prevalence of 56% for microcytic anaemia to be a common finding in the children examined when compared with 7.7% at follow-up. Microcytic anaemia, a surrogate marker of iron deficiency, is also a characteristic feature of anaemia due to malaria [33]. The anaemia of *P falciparum* malaria is typically normocytic and normochromic, with a notable absence of reticulocytes, although microcytosis and hypochromia may be present due to the very high frequency of alpha- and beta-thalassaemia traits and/or iron deficiency in many endemic areas [34]. However, Premji *et al*. [35] reported a majority of children infected with *P. falciparum* to be iron deficient. In a related study in the same area, 28% of the children with *P. falciparum* infections were iron deficient albeit the majority had microcytic anaemia [10]. It is therefore reasonable to state that the significant decline in microcytic anaemia observed in children at follow-up was the result of a reduction in prevalence of malaria parasitaemia. This consequently lends support to the fact that malaria is a major contributor to microcytic anaemia in the study population.

The primary limitation of the study is the lack of information on the variation in consultations with children under five years in health facilities following the commencement of free treatment of malaria as inferences have been made. However, irrespective of the age group, all the children benefitted in the same way from the environmental management instituted to control the breeding of the mosquito vector and the distribution of LLINs.

## Conclusions

The results from the studies suggest that even though a greater majority of the population is in possession of an ITN, less than half of the population use them effectively. Although malaria is still meso-endemic, most malariometric indices declined and the red cell indices markedly improved. Following interventions, anaemia (moderate to severe) was a more sensitive measure to detect changes in malaria exposure. Children in the 11–14 years age group experienced a significant increase in malaria-related morbidity, and microcytic anaemia was no longer a common finding in children in the area.

## Abbreviations

ACT: Artemisinin-based combination
AM: Asymptomatic malaria
CM: Clinical malaria
CI: 95% Confidence interval
GMPD: Geometric mean parasite density
Hb: Haemoglobin
Hct: Haematocrit
ITN: Insecticide-treated bed nets
LLIN: Long-lasting, insecticide-treated net
MA: Malarial anaemia
MCH: Mean corpuscular haemoglobin
MCHC: Mean corpuscular haemoglobin concentration
MCV: Mean corpuscular volume
NMCP: National Malaria Control Programme
RBM: Roll Back Malaria
RBC: Red blood cell
WBC: White blood cell.
